# Comment on Tagliaferro et al. Introducing the Novel Mixed Gaussian-Lorentzian Lineshape in the Analysis of the Raman Signal of Biochar. *Nanomaterials* 2020, *10*, 1748

**DOI:** 10.3390/nano13010108

**Published:** 2022-12-26

**Authors:** Robert J. Meier

**Affiliations:** Pro-Deo Consultant, 52525 Heinsberg, North-Rhine Westphalia, Germany; r.meier@planet.nl

Despite the fact that this comment deals with a paper that was published two years ago [1], it was only recently that the author of this comment became aware of it. As the fitting of spectra remains an important topic but also one in which many things are still frequently not well tackled, I considered that it still makes sense to submit this comment. To the knowledge and experience of the author, Tagliaferro et al. have made a number of correct initial statements; however, the translation into spectral fitting is seriously questioned here.

The key topic in this paper is the fitting of the line shape of a Raman spectrum of Biochar. In the words of Tagliaferro et al. in the abstract of their paper, ‘The individual contributions to the Raman spectra are difficult to identify due to the numerous peaks that contribute to the spectra’. In addition, ‘To tackle this problem, we propose a brand-new approach based on the introduction and on sound theoretical grounds of a mixed Gaussian—Lorentzian lineshape’. In addition, Tagliaferro et al. state, ‘In the present paper we will show that by relaxing on strong physical bases, the constraint of having a Gaussian lineshape, biochar Raman spectra can be decomposed by using two main peaks that can be readily identified as the usual D and G contributions’. Hence, whereas the authors speak of sound theoretical grounds and of a strong theoretical basis, later on in the paper we read, ‘we introduce a specific lineshape in the frequency domain that is able to account for all physical facts although not being yet fully justified on theoretical grounds’. Moreover, the fitting was not done using two main peaks identified as the usual D and G contributions, but by at least two for each of them (see e.g., Figure 6 in the paper). All this sounds somewhat contradictory within the paper, particularly with respect to the physical soundness.

The new line shape which was proposed is symmetrical with respect to its central wavenumber ω_0_, whereas its central part is Lorentzian, and the tails are Gaussians. In addition, the authors have applied a constraint: ‘We apply the further constraint that the curve must be of class C1 (i.e., both the functions and the derivatives must be continuous) in the transition points’, which is indeed what should be in a spectral feature. However, it remains obscure what is the physical basis for the point in the curve where the Lorentzian shape goes over into the Gaussian form, as now it is only based on the condition of continuity. Secondly, the two transition points are symmetrical with respect to the peak wavenumber, but why should they be for a Raman originating from an amorphous material with many different local environments? In the spectra shown by the authors, Figures 1 and 6, the 1355 cm^−1^ band looks very symmetrical to the naked eye, but this is, e.g., different for the spectrum shown by Tuinstra and Koenig, Ref. 49 in Tagliaferro et al. For other examples, see https://www.peaklab.net/peaklab-reports/raman-characterization-of-biochar-bonding/ (accessed on 17 August 2022). So how generic is their proposal for Biochar?

The argumentation that the new line shape named GauLor with Lorentz character in the central part and Gaussian wings is correct and is based on a comparison with the line shape resulting from a stretched exponential decay. Apart from the fact that it was not shown that stretched exponential decay is the correct mechanism here, the GauLor shape is a non-physical shape glued together from two shapes which, individually, do have a physical basis. In this context, it is interesting to note that it has been illustrated before [2] that the 80% upper part of the line shape is no indication for the line shape being Lorentzian or Gaussian: with a slightly different bandwidth selected, the Lorentz and Gaussian profiles fully overlap, except the bottom 20%. This means we can have a pure Gaussian profile which very closely reflects the Lorentzian profile in the central part with wings that are purely Gaussian, the shape the authors were looking for, and it is a profile that has physical justification. So, is there a proper basis for the GauLor choice?

Regarding the fitting of the spectra of Biochar, the authors have adopted multiple peaks to fit the experimental spectra (see their Figure 6) and noted ‘As a first step we define, on a meaningful physical basis, the number N of GauLor components we use for the spectral decomposition’. However, nowhere do they elaborate on what they called a meaningful physical basis. The authors make very strong statements as to their approach, and according to their initial statements, others have done the fitting incorrectly, but fitting 4–5 components under a broad spectral which exhibits only two visible maxima, such strong statements require strong proven facts justifying the additional 2–3 bands, e.g., by knowledge about specific species present and independent numerical data (e.g., from quantum calculations) on the position of such bands. Finally, Spectrum (b) OSR900 in Figure 6 exhibits an extremely broad band, the dark blue curve and nowhere is its width explained or justified. Taking 4–5 components and allowing for very broad bands, one can always fit the spectrum, but there is no a priori physical meaning behind this. The authors mention a D^1^ and G^2^ peak at some stage, but, to me, these are dropped somewhat from the sky without a clear discussion and reference to the literature, e.g., the D’ is well-known from the literature. Moreover, the background issue (see next paragraph) can severely modify the detailed outcome [2,3].

A very relevant issue is related to the way Tagliaferro et al. have drawn the background. For instance, looking at their Figure 6, we can see that the background line touches the experimental Raman spectral at the far left and the far right in each of the spectral plots, whereas the correct procedure is to take a spectral range clearly extending beyond the visible tails of the Raman bands and subsequently simultaneously fitting the bands and the background [2,3]. Taking this range to the left and the right is too restrictive and actually favours Gaussian line shapes compared to Lorentzian character and can thus lead to incorrect assessment of the true line shape. This is exactly the subject of the paper by Tagliaferro et al., namely that they support Gaussian tails whereas the background fitting procedure remains to be re-evaluated.

Moreover, various spectra that can be found in the literature reveal very broad D band spectral shapes, and therefore require taking account of a large spectral range to fit the Raman bands. This also brings us to Figure 9 in their paper where the authors argue that the fitting by a Gaussian or Lorentzian line shape requires many more components compared to their new line shape, 3–7 components are mentioned for the various spectra. In Figure 6, these were 4–5. The authors mention a D^1^ and G^2^ peak here, but to me these dropped somewhat from the sky without a clear discussion and reference to the literature, e.g., the D’ is well-known from the literature. 

Let us now get back to the original problem. Taglaiferro et al. have noted that, ‘The individual contributions to the Raman spectra are difficult to identify due to the numerous peaks that contribute to the spectra’. They also state, ‘On the other hand, if the local environment of the same oscillator type at different locations of the solid is different, the sum up of all contributions leads to a different lineshape’. These are fully correct statements. Apart from the issues we already mentioned, disordered materials are obviously more difficult than well-ordered materials. First of all, very well-ordered materials have sharp lines in the X-ray diffraction pattern, which is also reflected in the Raman spectra. However, Raman spectra may show relatively narrow lines due to local order, whereas X-ray diffraction data do not reveal any order. The difficulty, as one could say it, is that disordered material is not simply disordered material. In a small molecule liquid, the molecules are disordered, but they all have the same local (time-averaged) environment. For example, amorphous polymer one has a larger variety of environments, is basically frozen-in in a sense (time-scale Raman), and this can be associated with a band profile which is not symmetric. The way this could, in certain cases, be best fitted is to take the experimental band shape and use that, in numerical form, in the fitting procedure [4]. 
